# Bi-parametric MRI/TRUS fusion targeted repeat biopsy after systematic 10-12 core TRUS-guided biopsy reveals more significant prostate cancer especially in anteriorly located tumors

**DOI:** 10.1177/20584601221085520

**Published:** 2022-03-31

**Authors:** Michael Häggman, Pär Dahlman, Mats Ahlberg, Per Liss, Rafaele Cantera Ahlman, Anca Dragomir, Sam Ladjevardi

**Affiliations:** 1 Department of Urology, 59592 Uppsala University Hospital, Uppsala, Sweden; 2 Department of Radiology, 8097 Uppsala University Hospital, Uppsala, Sweden; 3 Department of Pathology, 59592 Uppsala University Hospital, Uppsala, Sweden; 4 Department of Immunology, Genetics and Pathology, 59592 Uppsala University, Uppsala, Sweden

**Keywords:** Prostate cancer, MRI, Bi-parametric MRI, fusion guided biopsy, transrectal ultrasound, diagnosis

## Abstract

**Background:**

MRI and fusion guided biopsy have an increased role in the diagnosis of prostate cancer.

**Purpose:**

To demonstrate the possible advantages with Bi-parametric MRI fusion-guided repeat biopsy over systematic 10–12-core biopsy for the diagnosis of prostate cancer.

**Material and Methods:**

Four hundred and twenty-three consecutive men, with previous systematic 10–12-core TRUS-guided biopsy, and with suspicion of, or diagnosis of, low-risk prostate cancer underwent fusion-guided prostate biopsy between February 2015 and February 2017. The material was retrospectively assessed. In 220 cases no previous cancer was diagnosed, and in 203 cases confirmatory fusion guided biopsy was performed prior to active monitoring. MRI was classified according to PI-RADS. Systematic biopsy was compared to fusion guided biopsy for the detection of cancer, and PI-RADS was compared to the Gleason score.

**Results:**

Fusion guided biopsy detected significantly more cancers than systematic (*p* < .001). Gleason scores were higher in the fusion biopsy group (*p* < .001). Anterior tumors were present in 54% of patients. Fusion biopsy from these lesions showed cancer in 53% with previously negative biopsy in systematic biopsies and 66% of them were upgraded from low risk to intermediate or high-risk cancers.

**Conclusion:**

These results show superior detection rate and grading of bi-parametric MRI/TRUS fusion targeted repeat biopsy over systematic 10–12 core biopsies. Fusion guided biopsy detects more significant cancers despite using fewer cores. The risk group was changed for many patients initially selected for active surveillance due to upgrading of tumors. Bi-parametric MRI shows promising results in detecting anterior tumors in patients with suspected prostate cancer.

## Introduction

Prostate cancer (PCa) remains one of the most diagnosed cancers among men worldwide.^1^ The diagnostic accuracy is still a challenge for both physician and the patient. An accurate verification of significant and potentially lethal PCa, excluding insignificant PCa poses a clinical problem. The prognosis of newly diagnosed PCa is dependent on risk group categorization. The most utilized system is the three-tier (low, intermediate, and high risk) D´Amico classification, mainly based on the histopathological grading of biopsies.^2^ The Gleason score has been the mainstay in histopathological grading of PCa. In 2005, the International society of Uropathology revised and clarified the Gleason score into the ISUP classification, with the 5-tier ISUP grading.^3^

It is well known that PSA testing and subsequent 10–12 core systematic biopsy leads to both overdiagnosis of clinically insignificant cancer and a risk of missing significant cancer.^4^ The systematic biopsies generally sample the dorsal part of the prostate. Even if 60–70% of tumors are in the peripheral zone,^5–7^ a substantial number risk being missed. A significant number of cases with negative systematic biopsies are later shown to harbor PCa and many PCa diagnosed by systematic biopsy are inaccurately graded. Each biopsy occasion may also lead to complications. In Sweden, 6% of patients are treated with antibiotics for urinary tract infections after prostate biopsies and cases with resistant bacteria become increasingly common.^8^

MRI plays a crucial role in identifying men with a high likelihood of clinically significant PCa who require immediate biopsy. Multiparametric MRI (mpMRI) has been shown to be a valuable tool to achieve more accurate biopsy sampling.^9^ It has also been shown that mpMRI may avoid diagnosing insignificant cancer, which prevents unnecessary biopsies, possibly reducing the side effects.^10^ Since more than 20% of the tumors are in the anterior part of the prostate,^11,12^ targeted biopsies may diagnose lesions in the prostate that are not diagnosed with systematic biopsies.^13^ However, it has been shown that targeted biopsies can miss significant cancer in some cases, and the sensitivity of this method needs improvement before it can replace systematic biopsies.^14^ A recent randomized study has shown the clear superiority of fusion-guided mpMRI/transrectal ultrasound (TRUS) biopsies over standard TRUS-guided biopsy.^15^

The added value of dynamic contrast-enhanced (DCE) MRI in combination with T2-weighted imaging and diffusion-weighted imaging (DWI) is controversial.^16^ The role of DCE MRI was recently downgraded by the American College of Radiology (ACR) and the European Society of Urogenital Radiology (ESUR) in the updated Prostate Imaging Reporting and Data System (PI-RADS) version 2.1. In detail, DWI for the peripheral zone (PZ) and T2-WI for the transition zone (TZ) were respectively considered the dominant sequences to detect clinically significant tumors. In PI-RADS v2, the role of DCE is minor, limited to potentially upgrading a PI-RADS three lesion in the PZ to PI-RADS 4.^17^

Nevertheless, in studies of men under active surveillance or those with a prior negative biopsy, the cancer detection rate approaches 55% and up to 94% in patients with highly suspicious lesions on MRI.^18^

It has been shown that the diagnostic accuracy of a bi-parametric MRI (bpMRI) imaging protocol, consisting of T2-weighted imaging and DWI, is comparable with that of a standard multi-parametric imaging protocol for the detection of clinically significant PCa.^19^

Initially, fusion between mpMRI and ultrasound (US) was done as “cognitive,” that is, the examiner intuitively calculated the position of the lesion to be biopsied and aimed the needles towards it under US visualization.

Subsequently, software for fusion by computer processing was developed, as a rigid, and at a later stage, elastic fusion. Rigid image registration overlays the MRI images onto the TRUS images during the biopsy procedure, without adjustment for possible deformation of the prostate caused by patient movement or the introduction of the TRUS probe.^20^ Elastic registration aims to compensate for this deformation, and it is therefore expected to be more accurate than rigid image registration.^21–23^

The aims of this study were to evaluate our adaption of bpMRI/TRUS fusion guided biopsy into clinical practice, to confirm previous reports that repeat MRI/TRUS fusion guided biopsy yields more significant (ISUP≥2) PCa as compared to initial systematic 10–12 core TRUS-guided biopsy, and third to study the distribution of anterior versus posterior tumors in this selected repeat biopsy cohort.

## Material and methods

Inclusion criteria for this retrospective study were men with diagnosed PCa with at least one set of systematic biopsies or men with suspicion of PCa defined as elevated PSA and/or other clinical findings giving rise to suspicion. The patients were either considered for active monitoring or had strong suspicion of more malignant tumor, due to discordance between the PSA level and Gleason score (GS) in systematic biopsies, that is, a high PSA value with only ISUP 1 tumor.

**Figure 1.**
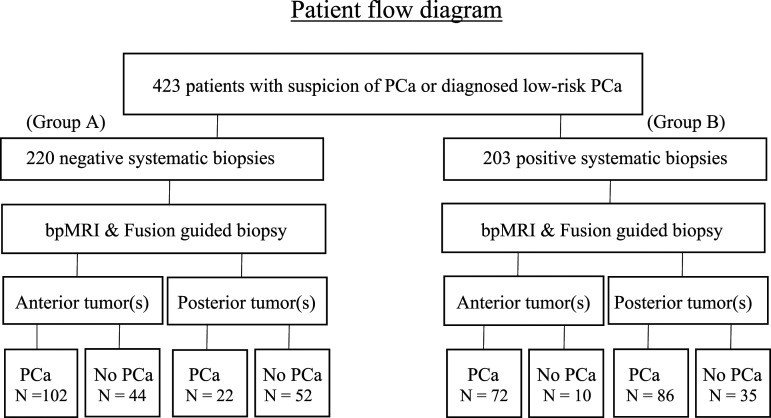
Patient flow diagram.

Between February 2015 and February 2017, all 423 consecutive men, aged 39–78, median 66 with mean PSA 14,3 (SD 1.9–63) undergoing bpMRI/TRUS fusion guided biopsy at our institution were included (Figure 1). Before the patients came to our institution, they had undergone one to seven systematic biopsy sets, predominantly three. At least two to 3 months had elapsed due to processing and evaluation of referrals before MRI examination and fusion-guided biopsy was performed. Data on age, MRI re-evaluation according to PI-RADS v. 2.0, PSA-value, clinical T-stage, number of and results from earlier systematic TRUS biopsies, treatment with Finasteride, and histopathological results were collected. Since ISUP grade is increasingly utilized worldwide, we classified histopathology results both by GS and ISUP grade.

Altogether 220 men had no known PCa prior to fusion biopsy (Group A). All of them had undergone 1–5 sets of prior negative 10–12 core systematic biopsies. Two hundred and three men had low-risk PCa (Gleason score 5–6 or ISUP 1) according to prior biopsies (Group B) and were candidates for active surveillance of their PCa.

### Research ethics and patient consent

The planned study was subjected to review and approved by the Regional Ethical Board (Uppsala Dnr 2012-165 with amendment 2016 and 2019-00534) and conducted in accordance. It was judged by The Regional Ethical Board that no informed consent was needed for this retrospective study.

### MRI assessment

All patients underwent MRI following the European Society of Urogenital Radiology (ESUR) guidelines; T2 weighted (axial, coronal, and sagittal), T1 with large field of view, apparent diffusion coefficient (ADC) and high trace value (b1000–b2000 s/mm2). Patients examined in house (55%) underwent bi-parametric 3T MRI (MRI scanner (Achieva, Philips Healthcare, Best, The Netherlands), high trace value b2000. Patients from other hospitals (45%) underwent 1.5 T (75%) (Siemens Aera and Philips) or 3T MRI (25%) (Siemens and Achieva). Before the MRI fusion-guided biopsy all MR examinations were re-evaluated and judged to fulfilled quality criteria. Suspected areas were assigned PI-RADS classification according to PI-RADS v. 2.0. One or more regions of interest (ROI) were delineated for fusion biopsy and segmentation data was transferred to the Artemis system. Two uroradiologists with 15 and 20 years of experience (PD and PL) were involved in the primary evaluating the studies at our own institution (55%, *N* = 233) and in re-evaluating all MRI studies from external institutions (45%, *N* = 190). In 90% of the evaluation of the MR examinations the evaluation was done by one of the two radiologists.

### Systematic biopsy

All patients had undergone one to five sets of systematic 10–12 core TRUS guided, side fire biopsies which were done “free hand,” that is, with a hand-held TRUS probe, by various physicians at our hospital or at referring centers.

### Fusion biopsy

We performed the fusion biopsy with the Artemis device (Artemis; Eigen, Grass Valley, CA), which allows biopsy site tracking on ultrasound and fusion of real-time ultrasound with MRI and the BK medical ultrasonography probe 8818 in end-fire mode. With the Artemis device the position of the US probe is tracked by angle-sensing devices (encoders), built into each joint of the probe-holding arm. This allows for the reconstruction of the biplanar US into a 3D model, which is then elastically fused with the MRI. Two to seven fusion guided biopsy cores at the examiners’ discretion (i.e., number and size of lesion(s), satisfaction with biopsy tracking, and patient compliance) were obtained from 1–3 ROI’s in all cases. All fusion biopsies were performed by two experienced urologists (SL and MH). Biopsies were processed as per standard protocol at our Pathology Department and examined by a senior, board-certified uropathologist with extensive experience of PCa pathology (AD).

### Statistical methods

MRI images were stratified into groups according to PI-RADS in “2–3” and “4–5.” For statistical analysis, Chi-squared 2 test with 95% confidence interval were used.

Cross tables were made comparing the number of individuals with no PCa for comparison of the groups and different GS detected by systematic biopsy and fusion biopsy. Sign test (exact binomial tests) was used to test if the number of detected PCa differed between standard biopsy and fusion biopsy and if the number of individuals with GS ≥ 7 differed between standard biopsy and fusion biopsy.

A comparison of GS for individuals with detected PCa at standard biopsy was done using the sign test.

Cross tables comparing PI-RADS score with GS from standard biopsy and GS from fusion biopsy were produced. The Spearman rank correlation between PI-RADS and GS for standard biopsy and fusion biopsy were assessed.

## Results

In group A, out of 220 patients with previous negative systematic biopsy, fusion biopsy diagnosed PCa in 124 (56%). In group B, among the 203 patients with previous low risk PCa after systematic biopsy, fusion biopsy detected cancer in 158 patients (78%) (Tables 1 and 2). The median number of ROI was 2^1–3^ for all 423 patients and the median number of Fusion guided biopsy cores was 5. Out of 423 patients, clinically significant (GS≥ 7, ISUP≥2) PCa was detected in 190 (45%) patients (Table 2).

**Table 1.** Baseline characteristics. Number of patients with undetected and detected prostate cancer in standard biopsy versus fusion biopsy.

Standard biopsy	Fusion biopsy			Sign test
	No PCa	PCa	Tot	*p*-value
No PCa	96	124	220	<.001
PCa	45	158	203	
Total	141	282	423	

*N* = 423.

**Table 2.** Gleason score in systematic biopsy compared to Gleason score in fusion biopsy.

Systematic biopsy	Fusion biopsy					
	No PCa	Gleason 6	Gleason 7a	Gleason 7b	Gleason 8	Gleason 9
No PCa	96 (23%)	34 (8%)	56 (13%)	23 (5%)	10 (2%)	1
Gleason 6	35 (8%)	43 (10%)	47 (11%)	15 (4%)	8 (2%)	0
Gleason 7a	9 (2%)	12 (3%)	19 (4%)	6 (1%)	2	0
Gleason 7b	1	3	1	0	0	0
Gleason 8	0	0	0	2	0	0

*N* = 423.

In group A, fusion biopsy detected intermediary risk (ISUP 2-3) PCa in 56 patients and high risk (ISUP 4-5) PCa in 34 patients. In group B, 62 patients were re-classified after fusion biopsy as intermediary risk (ISUP 2-3) and eight patients as high-risk cancer (ISUP 4-5). Among 27 patients initially diagnosed with intermediary risk cancer (ISUP 2-3) after systematic biopsy, eight patients were re-classified after fusion biopsy as high risk (ISUP 4-5) (Table 2). Upgrading in GS after fusion biopsy occurred in 202 cases, 158 were in concordance with systematic biopsies. In 63 cases after fusion biopsy the GS was downgraded (*p* < .001), (Table 3).

**Table 3.** A comparison of severity obtained using systematic biopsy versus fusion biopsy. Tested using a sign test. (Severity ordered as No PCa, GS 6, GS 7a, GS 7b, GS 8, and GS 9).

Comparison of severity from biopsy			Sign test
Higher in standard	Equal	Higher i fusion	*p*-value
63	158	202	*<*.001

*N* = 423.

In total, 51 patients were classified as PI-RADS 2 and underwent fusion biopsy. These patients were included in the initial phase of the study, when our experience was limited, and during that phase all PI-RADS 2-5 lesions were targeted with fusion biopsy. It was soon realized that the diagnostic yield of PI-RADS two lesion was limited, as noted elsewhere in clinical practice. Since the mentioned PI-RADS 2 cases fulfilled the inclusion criteria we decided to include them in the analysis of the 423 consecutive cases. Those patients had an average PSA of 10.6 ng/mL (range 3–54) which does not differ from the total material. Seventeen (33%) of 51 patients had a previous PCa-diagnosis after systematic biopsies, 11 patients (22%) with GS 6 (ISUP 1), and six patients (12%) with GS 7-8 (ISUP 2-4). Fusion biopsies revealed PCa in nine patients (18%), of which 5 (10%) had GS 6 (ISUP1), 3 (6%) had GS 7 (ISUP2-3), and one (2%) had GS 8 (ISUP4) (Tables 4 and 5).

**Table 4.** PIRADS score versus Gleason score using systematic biopsy. Spearman rank correlation: 0.103.

PIRADS	Systematic biopsy					
	No PCa	Gleason 6	Gleason 7a	Gleason 7b	Gleason 8	Total PCa
2	34 (8%)	11 (3%)	5 (1%)	0	1	17
3	74 (17%)	46 (11%)	12 (3%)	0	1	59
4	77 (18%)	62 (15%)	26 (6%)	5 (1%)	0	93
5	35 (8%)	29 (7%)	5 (1%)	0	0	34
Total	220	148	48	5	2	203

*N* = 423.

**Table 5.** PIRADS score versus Gleason score using fusion biopsy. Spearman rank correlation: 0.488.

PIRADS	Fusion biopsy						
	No PCa	Gleason 6	Gleason 7a	Gleason 7b	Gleason 8	Gleason 9	Total PCa
2	42 (10%)	5 (1%)	1	2	1	0	9
3	59 (14%)	31 (7%)	33 (8%)	9 (2%)	1	0	74
4	38 (9%)	47 (11%)	56 (13%)	18 (4%)	10 (2%)	1	132
5	2	9 (2%)	33 (8%)	17 (4%)	8 (2%)	0	67
Total	141	92	123	46	20	1	282

*N* = 423.

One hundred and thirty-three patients were classified as PI-RADS 3. For 59 (44%) of them PCa was diagnosed by systematic biopsy, with GS 6 (ISUP1) for 46 (35%) patients, GS 7a (ISUP2) for 12 (9%) and GS 8 (ISUP4) for 1. After fusion biopsies PCa was found in 74 (56%) patients, of which 31 (42%) had GS 6 (ISUP1), 33 (45%) had GS 7a (ISUP2) and 9 (12%) GS 7b (ISUP3), and one with GS 8 (ISUP 4) (Tables 4 and 5).

One hundred and seventy patients were classified as PI-RADS 4. Previous systematic biopsies had shown PCa, in 93 (55%) of patients: 62 (36%) with GS 6 (ISUP1), 26 (15%) with GS 7a (ISUP2), and 5 (3%) with GS 7b (ISUP3). After fusion-biopsies PCa was found in 132 (77%) patients: 47 (28%) had GS 6 (ISUP1), 56 (33%) had GS 7a (ISUP2), 18 (11%) had GS 7b (ISUP3), 10 (6%) had GS 8 (ISUP4), and 1 had GS 9 (ISUP5). Two patients had no specified GS due to Finasteride treatment.

Sixty-nine patients were classified as PIRADS 5 and 34 (49%) of them had prior PCa-diagnosis after systematic biopsies: 29 (42%) with GS 6 (ISUP1), 5 (7%) with GS 7a (ISUP2). After fusion biopsies PCa was found in 67 patients: 9 (13%) with GS 6 (ISUP1), 33 (48%) with GS 7a (ISUP2) and 17 with GS 7b (24%) (ISUP3), 8 (12%) with GS 8 (ISUP4) (Tables 4 and 5).

In group A, out of 220 cases, 34 (15%) were upgraded from no cancer to low risk and 90 (41%) to intermediate/high risk cancer by repeat fusion-guided biopsy. In group B, out of 203 cases, 70 (34%) changed from low risk to intermediate/high risk cancer. Thus, in total 160 cases had their risk group upgraded, possibly to be subjected to curative treatment (Table 6).

**Table 6.** Possible changes in management of PCa as caused by repeat fusion biopsy upgrading. Change in risk group from low to intermediate will greatly influenced treatment decisions.

No PCa	Low risk PCa (ISUP 1)	Intermediate/High risk PCa (ISUP 2–5)
220 (Group A)	34 (15%)	
220 (Group A)		90 (41%)
203 (Group B)		70 (34%)

*N* = 423.

**Figure 2.**
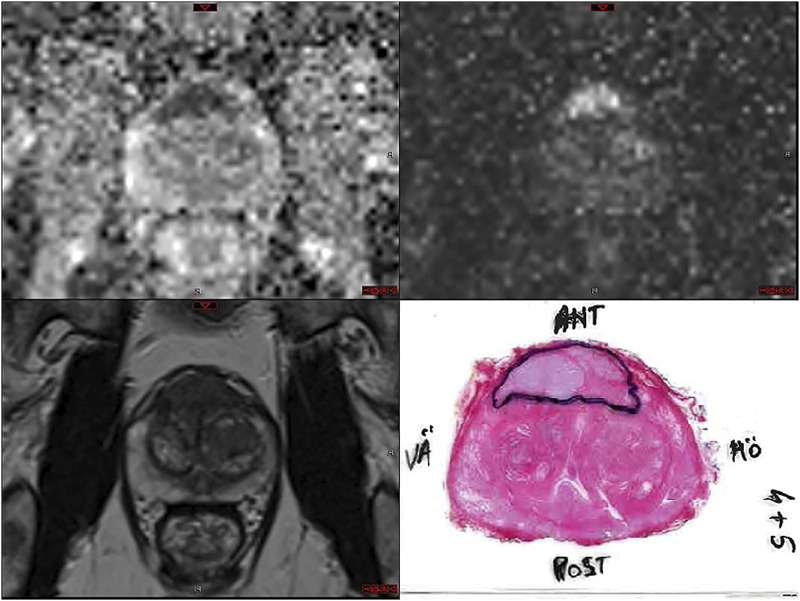
MRI images (ADC, DWI, T2) and corresponding histopathological whole-mount slide of radical proctectomy specimen.

Among all patients who were included in this study bpMRI revealed 228 (54%) patients with anterior tumors and 195 (46%) patients with posteriorly located tumors**.** Anterior tumors were larger than posterior tumors. The average anterior and posterior tumor diameter on MRI was 1.8 cm (SD = 0.69) and 1.0 cm (SD = 0.48), respectively. The difference was mainly due to there being few large posterior tumors. (Figure 2). In group A, 146 patients (66%) had anterior tumors on bpMRI. Of those, 102 (70%) patients had cancer, whereof 78 patients (53%) had intermediate/high risk cancer at fusion biopsy. In group B, bpMRI revealed 82 (40%) patients with anteriorly located tumor. Of those, 54 (66%) were upgraded to intermediate/high risk cancer at fusion biopsy. In 10 patients with anterior tumors when systematic biopsies diagnosed GS 3+3 (ISUP 1) in insignificant amounts (less than 3 mm of cancer), fusion biopsy failed to find cancer. These patients remain on active monitoring. In another 44 patients with anterior tumors, both systematic and TRUS/fusion biopsy were negative, and these patients were not further investigated (Table 7).

**Table 7.** Gleason scores in anterior tumors.

Fusion biopsy	Previously negative systematic biopsy	Previously low risk systematic biopsy		
		GS 3+3	GS 3+4	
No PCa	44	10	0	54 (22%)
GS 3+3	24	18	0	42 (18%)
GS 3+4 (7a)	39	35	5	79 (35%)
GS 4+3 (7b)	23	8	2	33 (14%)
GS ≥ 8	16	4	0	20 (9%)
	146	75	7	228

*N* = 228.

## Discussion

Our main findings in this study, analyzing our results after implementing bpMRI-TRUS fusion biopsies at our institution in February 2015, is that bpMRI-TRUS fusion biopsies show superior detection rate and grading than systematic 10–12 core biopsies, fusion guided biopsy detects more significant cancers despite using fewer cores, the risk group was changed for many patients from active surveillance to upgrading of tumors and better detecting of anterior tumors in patients with suspected prostate cancer. These results are comparable to previously published reports.

Prostate cancer (Gleason score ≥6) was detected by fusion biopsy in 67% of the patients in our cohort, and thus bpMRI and our Artemis TM software augmented method is feasible in clinical practice. Histopathological upgrading and new cancers diagnosed by repeat fusion biopsy after systematic biopsies had substantial impact on risk categories in our material. The management of patients was influenced by the repeat fusion biopsies in 160/423 (38%) of the cases. In group A, 90 (41%) had intermediate/high risk cancer diagnosed and were considered for curative treatment. In group B, 70 (34%) patients were upgraded and fulfilled the criteria for curative treatment.

MRI has an increasingly important role in PCa diagnostics and is recommended in men with previously negative TRUS biopsy. In some national guidelines MRI is now recommended prior to biopsy.^24^ The optimal biopsy method after MRI is under discussion. Wegelin et al.^25^ showed in a systematic review that In-bore versus MRI/TRUS-guided fusion versus Cognitive Fusion that none of these techniques were able to demonstrate significant differences between fusion biopsy and cognitive biopsy on overall cancer and clinically significant cancer detection. The PROMIS study was performed to test the diagnostic accuracy of mpMRI and systematic TRUS biopsy against template mapping biopsy in biopsy-naive patients. Upfront mpMRI would exclude 27% of cases from primary biopsy and reduce overdiagnosis of clinically insignificant PCa, while diagnosing more cases of clinically significant PCa.^26^ In our study, all patients who were already diagnosed with significant posterior tumors on systematic biopsy beforehand were not eligible.

We have chosen to use bpMRI instead of mpMRI, the latter being more time consuming and expensive than bpMRI. Use of gadolinium contrast exposes patients to further risks, for example, allergic reactions, risk of NSF (nephrogenic systemic fibrosis), and depositions in the brain.^27–29^

It has been demonstrated that the sensitivity of bpMRI in the detection of PCa is similar to that of mpMRI and some investigators have found that Dynamic contrast enhancement could potentially increase the false positive rate.^30,31^ Woo et al.^32^ conclude that bpMRI has equal diagnostic performance to mpMRI for the detection of PCa. The consensus among experienced prostate radiologists in the ESUR prostate working group is that experienced radiologists who work closely with urologists and receive continuous feedback perform equally well on mpMRI or bpMRI.

The cancer detection rate increased with increasing PI-RADS score. We identified 184 patients with PI-RADS 2-3. Among these we found 83 (45%) patients with positive fusion guided biopsies of which 36 (20%) patients were diagnosed with GS 6 (ISUP 1). It has been shown in other studies that PI-RADS two lesions most likely do not represent clinically significant PCa.^33^ Therefore, later during the study, we chose PI-RADS 3 as cut-off. Seventeen (33%) of the 51 cases with PI-RADS two lesions had prior positive systematic biopsies. Fusion guided biopsy revealed fewer cancers, nine (18%) than the systematic biopsies. This important observation made us discontinue biopsying PI-RADS two lesions for the remainder of the study period. However, for reasons mentioned previously, we decided not to exclude the initial 51 PI-RADS 2 cases. The fusion technique reduced the number of biopsies done on patients who had low-grade PCa, thus increasing the detection of the intermediate and high-risk subgroups of patients compared to traditional modalities.^34^ Our study shows the same results.

Targeted biopsy yielded a significantly higher positive rate for biopsy cores, and more importantly, more significant PCa was detected.^34–36^ Our observation is in line with previous observations that repeat fusion guided biopsy gives a higher yield of positive biopsies, and higher proportion of tumor infiltration in each core, compared to repeat systematic biopsies.^34,37^ The lesser number of cores needed for fusion guided biopsy decreases the risk of biopsy related complications.

The location of the tumor within the prostate has a significant impact on the diagnostic yield of systematic 10-12 core biopsies. These cores predominantly sample the posterior part of the gland and thus there is a risk of missing significant lesions in the anterior parts.^12,38,39^ MRI can identify anterior lesions and fusion-guided targeted biopsy will diagnose cancer in a significant number of lesions as shown in our study. Most of the tumors in our study were located anteriorly (54%). In this cohort of repeat biopsied patients, posterior intermediate/high-risk tumors are not present, since they were previously sufficiently diagnosed by systematic biopsy. Thus, the proportion of anterior tumors in our study is apparently higher than studies on biopsy-naive patients. Of 228 patients with anterior tumors, systematic biopsy revealed 82 (36%) patients with cancer. Fifty-four out of those (66%) were upgraded after MRI/TRUS-guided biopsy to intermediate/high risk cancer. These patients were later considered for curative therapy.

Urologists diagnosing PCa are aware of tumor heterogeneity and the importance of adequate sampling to appropriately assess the aggressiveness of the cancer. By utilizing a targeted approach to PCa detection, more tissue can be sampled from the lesions of interest as opposed to systematic biopsies, which would also sample benign or clinically insignificant lesions. Several studies have shown the benefit of utilizing MRI/TRUS fusion guided prostate biopsy in the diagnosis of PCa.^9,40^ Siddiqui et al. evaluated 1003 men undergoing both MRI/TRUS targeted biopsy and systematic biopsy. They showed that MR/TRUS fusion biopsy was associated with increased detection of high-risk PCa. Patients with PI-RADS 2-3 were mostly associated with detection of low-risk PCa. The same study demonstrated MR/TRUS fusion biopsy to better predict final pathology on subsequent radical prostatectomy.^41^

There are several weaknesses in our study. It is a retrospective study with known weaknesses. In some of the patients, detailed information regarding the number of previous biopsies was insufficient. There was no control group of patients who received a full multiparametric MRI protocol. The study was performed from all our first 432 MR/TRUS fusion biopsies which also include our initial patients with a natural learning curve. At the start, we chose to also biopsy PIRADS two changes which were stopped after 6 months when we felt more confident in our judgment. These initial biopsied PIRADS two changes are included in the material. As a tertial referral center, 45% (*N* = 190) of the MR examinations were done outside our institution and though all examinations were judged to fulfilled quality criteria it is of importance to bear in mind that the MR examinations are from several different institutions.

The higher rate of significant PCa in MRI/TRUS fusion guided biopsies supports primary MRI- fusion guided biopsy omitting systematic biopsies. This strategy is also supported by Rastinehad^9^ and Rouviere in a randomized trial.^13^

In conclusion, bpMRI/TRUS fusion guided repeat biopsy, both for primary diagnosis and follow-up biopsies in patients with small amounts of PCa on systematic biopsy significantly increases diagnostic yields particularly in anterior tumors. BpMRI/TRUS fusion guided biopsies provide better basis for therapeutic decisions compared to systematic biopsies.

## References

[1] Ferlay J Soerjomataram I Dikshit R , et al. Cancer incidence and mortality worldwide: sources, methods and major patterns in GLOBOCAN 2012. Int J Cancer 2015; 136: 359–386. 10.1002/ijc.29210 25220842

[2] D’Amico AV Whittington R Malkowicz SB , et al. Biochemical outcome after radical prostatectomy, external beam radiation therapy, or interstitial radiation therapy for clinically localized prostate cancer. JAMA 1998; 280: 969–974. 9749478 10.1001/jama.280.11.969

[3] Srigley JR Delahunt B Samaratunga H , et al. Controversial issues in Gleason and International Society of Urological Pathology (ISUP) prostate cancer grading: proposed recommendations for international implementation. Pathology 2019; 51: 463–473. 31279442 10.1016/j.pathol.2019.05.001

[4] Mpalang RKA Boreux R Melin P , et al. Prevalence of campylobacter among goats and retail goat meat in Congo. J Infect Dev Ctries 2014; 8: 168–175. 24518626 10.3855/jidc.3199

[5] McNeal JE Redwine EA Freiha FS , et al. Zonal distribution of prostatic adenocarcinoma. Am J Surg Pathol 1988; 12: 897–906. 3202246 10.1097/00000478-198812000-00001

[6] Reissigl A Pointner J Strasser H , et al. Frequency and clinical significance of transition zone cancer in prostate cancer screening. Prostate 1997; 30: 130–135. 9051151 10.1002/(sici)1097-0045(19970201)30:2<130::aid-pros8>3.0.co;2-s

[7] Kabalin JN McNeal JE Price HM , et al. Unsuspected adenocarcinoma of the prostate in patients undergoing cystoprostatectomy for other causes: incidence, histology and morphometric observations. J Urol 1989; 141: 1091–1093; discussion 3–4. 2785219 10.1016/s0022-5347(17)41178-5

[8] Lundström K-J Drevin L Carlsson S , et al. Nationwide population based study of infections after transrectal ultrasound guided prostate biopsy. J Urol 2014; 192: 1116–1122. 24813343 10.1016/j.juro.2014.04.098

[9] Valerio M Donaldson I Emberton M , et al. Detection of clinically significant prostate cancer using magnetic resonance imaging-ultrasound fusion targeted biopsy: a systematic review. Eur Urol 2015; 68: 8–19. 25454618 10.1016/j.eururo.2014.10.026

[10] Nam RK Saskin R Lee Y , et al. Increasing hospital admission rates for urological complications after transrectal ultrasound guided prostate biopsy. J Urol 2013; 189: S12–S18; discussion S7–8. 23234616 10.1016/j.juro.2012.11.015

[11] Bott SRJ Young MPA Kellett MJ , et al. Anterior prostate cancer: is it more difficult to diagnose? BJU Int 2002; 89: 886–889. 12010233 10.1046/j.1464-410x.2002.02796.x

[12] Kudlackova S Kurfurstova D Kral M , et al. Do not underestimate anterior prostate cancer. Biomed Pap Med Fac Univ Palacky Olomouc Czech Repub 2021; 165: 198–202. 33252117 10.5507/bp.2020.054

[13] Baco E Rud E Ukimura O , et al. Effect of targeted biopsy guided by elastic image fusion of MRI with 3D-TRUS on diagnosis of anterior prostate cancer. Urol Oncol 2014; 32: 1300–1307. 25189687 10.1016/j.urolonc.2014.07.014

[14] Kasivisvanathan V Rannikko AS Borghi M , et al. MRI-targeted or standard biopsy for prostate-cancer diagnosis. N Engl J Med 2018; 378: 1767–1777. 29552975 10.1056/NEJMoa1801993 PMC9084630

[15] Barentsz JO Richenberg J Clements R , et al. ESUR prostate MR guidelines 2012. Eur Radiol 2012; 22: 746–757. 22322308 10.1007/s00330-011-2377-y PMC3297750

[16] Vargas HA Hötker AM Goldman DA , et al. Updated prostate imaging reporting and data system (PIRADS v2) recommendations for the detection of clinically significant prostate cancer using multiparametric MRI: critical evaluation using whole-mount pathology as standard of reference. Eur Radiol 2016; 26: 1606–1612. 26396111 10.1007/s00330-015-4015-6 PMC4803633

[17] Bjurlin MA Mendhiratta N Wysock JS , et al. Multiparametric MRI and targeted prostate biopsy: Improvements in cancer detection, localization, and risk assessment. Cent Eur J Urol 2016; 69: 9–18. 10.5173/ceju.2016.734 PMC4846729 27123316

[18] Di Campli E Delli Pizzi A Seccia B , et al. Diagnostic accuracy of biparametric vs multiparametric MRI in clinically significant prostate cancer: comparison between readers with different experience. Eur J Radiol 2018; 101: 17–23. 29571792 10.1016/j.ejrad.2018.01.028

[19] Kaplan I Oldenburg NE Meskell P , et al. Real time MRI-ultrasound image guided stereotactic prostate biopsy. Magn Reson Imag 2002; 20: 295–299. 10.1016/s0730-725x(02)00490-3 12117612

[20] Rud E Baco E Eggesbø HB , et al. MRI and ultrasound-guided prostate biopsy using soft image fusion. Anticancer Res 2012; 32: 3383–3389. 22843919

[21] Natarajan S Marks LS Margolis DJA , et al. Clinical application of a 3D ultrasound-guided prostate biopsy system. Urol Oncol 2011; 29: 334–342. 21555104 10.1016/j.urolonc.2011.02.014 PMC3432280

[22] Verma S Choyke PL Eberhardt SC , et al. The current state of MR imaging-targeted biopsy techniques for detection of prostate cancer. Radiology 2017; 285: 343–356. 29045233 10.1148/radiol.2017161684 PMC5673043

[23] Ukimura O Desai MM Palmer S , et al. 3-Dimensional elastic registration system of prostate biopsy location by real-time 3-dimensional transrectal ultrasound guidance with magnetic resonance/transrectal ultrasound image fusion. J Urol 2012; 187: 1080–1086. 22266005 10.1016/j.juro.2011.10.124

[24] Bratt O Thellenberg C Carlsson S , et al. The Swedish National Guidelines on Prostate Cancer, Part 1: Early Detection, Diagnostics, Staging, Patient Support and Primary Management of Non-metastatic Disease. Scand J Urol. Accepted for publication in February 2022. 10.1080/21681805.2022.2094462 35811480

[25] Wegelin O van Melick HHE Hooft L , et al. Comparing three different techniques for magnetic resonance imaging-targeted prostate biopsies: a systematic review of in-bore versus magnetic resonance imaging-transrectal ultrasound fusion versus cognitive registration. Is there a preferred technique? Eur Urol 2017; 71: 517–531. 27568655 10.1016/j.eururo.2016.07.041

[26] Ahmed HU El-Shater Bosaily A Brown LC , et al. Diagnostic accuracy of multi-parametric MRI and TRUS biopsy in prostate cancer (PROMIS): a paired validating confirmatory study. Lancet 2017; 389: 815–822. 28110982 10.1016/S0140-6736(16)32401-1

[27] Obmann VC Pahwa S Tabayayong W , et al. Diagnostic accuracy of a rapid biparametric MRI protocol for detection of histologically proven prostate cancer. Urology 2018; 122: 133–138. 30201301 10.1016/j.urology.2018.08.032 PMC6295224

[28] Junker D Steinkohl F Fritz V , et al. Comparison of multiparametric and biparametric MRI of the prostate: are gadolinium-based contrast agents needed for routine examinations? World J Urol 2019; 37: 691–699. 30078170 10.1007/s00345-018-2428-y

[29] McDonald RJ McDonald JS Kallmes DF , et al. Intracranial gadolinium deposition after contrast-enhanced MR imaging. Radiology 2015; 275: 772–782. 25742194 10.1148/radiol.15150025

[30] Kuhl CK Bruhn R Krämer N , et al. Abbreviated biparametric prostate MR imaging in men with elevated prostate-specific antigen. Radiology 2017; 285: 493–505. 28727544 10.1148/radiol.2017170129

[31] Stanzione A Imbriaco M Cocozza S , et al. Biparametric 3T magentic resonance imaging for prostatic cancer detection in a biopsy-naïve patient population: a further improvement of PI-RADS v2? Eur J Radiol 2016; 85: 2269–2274. 27842676 10.1016/j.ejrad.2016.10.009

[32] Woo S Suh CH Kim SY , et al. Head-to-head comparison between biparametric and multiparametric MRI for the diagnosis of prostate cancer: a systematic review and meta-analysis. Am J Roentgenol 2018; 211: W226–W241. 30240296 10.2214/AJR.18.19880

[33] Liddell H Jyoti R Haxhimolla HZ , et al. mp-MRI prostate characterised PIRADS 3 lesions are associated with a low risk of clinically significant prostate cancer - a retrospective review of 92 biopsied PIRADS 3 lesions. Curr Urol 2015; 8: 96–100. 26889125 10.1159/000365697 PMC4748763

[34] Baco E Rud E Eri LM , et al. A randomized controlled trial to assess and compare the outcomes of two-core prostate biopsy guided by fused magnetic resonance and transrectal ultrasound images and traditional 12-core systematic biopsy. Eur Urol 2016; 69: 149–156. 25862143 10.1016/j.eururo.2015.03.041

[35] Maxeiner A Fischer T Stephan C , et al. Die echtzeit-MRT/US-fusionsbiopsie verbessert die detektionsrate des prostatakarzinoms nach mehrfach negativen Vorbiopsien. Aktuelle Urol 2014; 45: 197–203. 24902069 10.1055/s-0034-1375682

[36] Rastinehad AR Turkbey B Salami SS , et al. Improving detection of clinically significant prostate cancer: magnetic resonance imaging/transrectal ultrasound fusion guided prostate biopsy. J Urol 2014; 191: 1749–1754. 24333515 10.1016/j.juro.2013.12.007 PMC8374473

[37] Boesen L Nørgaard N Løgager V , et al. Multiparametric MRI in men with clinical suspicion of prostate cancer undergoing repeat biopsy: a prospective comparison with clinical findings and histopathology. Acta Radiol 2018; 59: 371–380. 28679325 10.1177/0284185117718400

[38] Maxeiner A Nest AM Stephan C , et al. Additive value of transrectal systematic ventral biopsies in combination with magnet resonance imaging/ultrasound fusion-guided biopsy in patients with 3 or more negative prostate biopsies. Urol Int 2020; 104: 205–213. 31801153 10.1159/000504266

[39] Porreca A Bianchi FM Salvaggio A , et al. Prognostic performance of magnetic resonance imaging-guided biopsy in defining prostate cancer anterior lesions. World J Urol 2021; 39: 1473–1479. 32621027 10.1007/s00345-020-03335-4

[40] Boesen L Nørgaard N Løgager V , et al. Where do transrectal ultrasound- and magnetic resonance imaging-guided biopsies miss significant prostate cancer? Urology 2017; 110: 154–160. 28866023 10.1016/j.urology.2017.08.028

[41] Siddiqui MM Rais-Bahrami S Turkbey B , et al. Comparison of MR/ultrasound fusion-guided biopsy with ultrasound-guided biopsy for the diagnosis of prostate cancer. JAMA 2015; 313: 390–397. 25626035 10.1001/jama.2014.17942 PMC4572575

